# Dataset of round-level federated learning and layer-2 blockchain overhead from ten raspberry Pi edge clients

**DOI:** 10.1016/j.dib.2026.113111

**Published:** 2026-07-25

**Authors:** Talgar Bayan, Bibarys Mukhambetiyar, Askar Boranbayev, Adnan Yazici

**Affiliations:** a Nazarbayev University, 53 Kabanbay Batyr Avenue, Astana 010000, Kazakhstan; b University of Manchester, Oxford Road, Manchester M13 9PL, United Kingdom

**Keywords:** Smart contract, On-chain provenance, Base mainnet, Decentralised storage, IPFS, Device telemetry

## Abstract

This data article describes round-level records from 40 federated learning sessions run on a physical testbed of ten Raspberry Pi 4 Model B devices acting as edge clients, with an Ubuntu workstation as the aggregation server. Each session trains a one-dimensional Squeeze-and-Excitation ResNet classifier over ten aggregation rounds using the Flower framework and commits every round to a smart contract on Base mainnet, a public Ethereum-compatible Layer-2 chain, while pinning the model and metric artifacts to IPFS through Pinata. The sessions cover two human activity recognition benchmarks, MHEALTH and UCI-HAR, and two middleware configurations: a baseline that performs blockchain and IPFS operations synchronously with per-round gas estimation, and an optimised variant that overlaps IPFS uploads with local training and reuses cached gas parameters, giving 20 sessions per benchmark. For every round the dataset records server-side global metrics (accuracy, F1, AUC, and the confusion matrix), per-device training telemetry (loss, training time, CPU, memory, and temperature), per-client evaluation records, and the IPFS content identifiers and transaction hashes that link each round to Base mainnet. For every session, millisecond-resolution logs record the latency of each blockchain and storage operation. The repository contains 12 CSV files, two archives of raw session directories, and a data dictionary. Researchers can reuse the convergence traces to benchmark edge federated learning, the device telemetry to characterise resource use on constrained hardware, and the latency distributions to parameterise simulations of blockchain-enabled federated learning without physical deployments or transaction fees. All 1240 blockchain transactions remain publicly verifiable on the Base mainnet explorer.

Specifications Table

**Table:** 

Subject	Computer Sciences
Specific subject area	Federated learning on edge hardware; on-chain auditing of training rounds on a public Layer-2 blockchain; human activity recognition.
Type of data	Table (.csv) — aggregated metrics, device telemetry, and transaction–pin maps Text (.json round-event records in newline-delimited JSON; plain-text .log performance traces) Image (.png confusion matrices) Raw; Analysed
Data collection	Records were produced by instrumented Flower 1.29 federated-learning server and client software running on ten Raspberry Pi 4 Model B devices connected over 2.4 GHz Wi-Fi to an Ubuntu aggregation server. web3.py 7.x middleware recorded every transaction on Base mainnet; the Pinata pinning API recorded IPFS pinning. During training and evaluation, each device recorded its CPU load and RAM with *psutil*, and its temperature from the Raspberry Pi thermal sensor. Two Python scripts (aggregate_metrics.py, extract_perf_timings.py) flatten the raw per-session logs into the deposited CSV tables; no value is imputed.
Data source location	Institution: Nazarbayev University City/Region: Astana Country: Kazakhstan Latitude and longitude: 51.0909, 71.4187 On-chain records: Base mainnet, contract 0xBE3FeAC76293711C5D32426860303AfE24cdf527 Primary source datasets used for training: MHEALTH (https://archive.ics.uci.edu/dataset/319/mhealth+dataset); UCI-HAR (https://archive.ics.uci.edu/dataset/240/human+activity+recognition+using+smartphones)
Data accessibility	Repository name: Mendeley Data DOI: https://doi.org/10.17632/dgmjr3ngtv.2 Direct URL to data: https://data.mendeley.com/datasets/dgmjr3ngtv/2 Instructions for accessing these data: download the twelve aggregated .csv files and the two raw session .zip archives; DATA_DICTIONARY.md documents every file, field, and value. Source code, aggregation scripts, and the smart contract are mirrored at https://github.com/bmukhambetiyar/FLBL2. Licence: CC BY 4.0.
Related research article	None

## 1 Value of the Data

• The dataset uniquely combines per-round federated learning performance metrics, device-level hardware telemetry, and real blockchain transaction overhead collected from a public production Layer-2 blockchain (Base mainnet) using physical Raspberry Pi 4 edge devices. Unlike studies based on simulations or permissioned blockchain environments, these data capture the practical costs and performance implications of on-chain auditing under realistic network, infrastructure, and hardware conditions [1,2].
• The millisecond-resolution timing logs (perf_timings_flowertrained_mhealth.csv and perf_timings_flowertrained_ucihar.csv) capture the latency of the main blockchain and storage operations. These empirical measurements can be used to parameterise and validate simulations of blockchain-enabled federated learning without physical hardware or transaction costs.
• The dataset includes both baseline and optimised configurations evaluated on the MHEALTH and UCI-HAR benchmarks under the same blockchain infrastructure. This allows researchers to compare synchronous and asynchronous middleware strategies, assess optimisation techniques, and separate dataset-specific effects from infrastructure-related impacts on training latency, communication overhead, and federated learning convergence [3].
• The dataset includes per-round convergence traces and device-level telemetry (training time, CPU utilisation, memory usage, and temperature), enabling researchers to benchmark federated learning on resource-constrained edge devices and analyse resource consumption, energy efficiency, and thermal behaviour during prolonged on-device training.
• All 1240 on-chain transactions are publicly accessible on the Base mainnet explorer; the tx_pin_map files link each round to a transaction hash, a Basescan URL, and an IPFS content identifier, letting any third party verify dataset integrity and provenance without contacting the authors [4].

## 2 Background

Federated learning enables multiple devices to collaboratively train a shared model without exchanging raw data, making it well suited for privacy-sensitive health and activity-recognition applications on edge and IoT devices [3,5]. Blockchain-based auditing adds a tamper-evident record of model updates and aggregation rounds [6], while IPFS provides efficient off-chain storage for model artifacts. However, most existing studies rely on simulated environments or permissioned blockchains and rarely report the real-world overhead of blockchain operations on resource-constrained hardware [1,2,7]. To address this gap, we developed a physical ten-node Raspberry Pi 4 testbed, recorded every federated learning round on Base mainnet and stored model artifacts on IPFS while instrumenting all blockchain and storage operations. The resulting dataset captures both federated learning performance and the infrastructure overhead of public blockchain auditing at round-level granularity. This data article documents the dataset and enables independent reuse of the convergence traces, device telemetry, and blockchain timing logs.

## 3 Data Description

The dataset is deposited on Mendeley Data as a flat collection of 15 files: 12 aggregated comma-separated (.csv) tables, a Markdown data dictionary (DATA_DICTIONARY.md), and two ZIP archives holding the raw per-session directories. The file layout is:

• DATA_DICTIONARY.md.
• fl_raw_results_mhealth.zip (20 MHEALTH session directories).
• fl_raw_results_ucihar.zip (20 UCI-HAR session directories).
• all_rounds_flowertrained_mhealth.csv (220 rows).
• all_rounds_flowertrained_ucihar.csv (220 rows).
• all_devices_flowertrained_mhealth.csv (2000 rows).
• all_devices_flowertrained_ucihar.csv (2000 rows).
• all_client_evals_flowertrained_mhealth.csv (2000 rows).
• all_client_evals_flowertrained_ucihar.csv (2000 rows).
• perf_timings_flowertrained_mhealth.csv (2560 rows).
• perf_timings_flowertrained_ucihar.csv (2560 rows).
• session_summary_flowertrained_mhealth.csv (20 rows).
• session_summary_flowertrained_ucihar.csv (20 rows).
• tx_pin_map_flowertrained_mhealth.csv (840 rows).
• tx_pin_map_flowertrained_ucihar.csv (840 rows).

The dataset comprises twelve aggregated tables generated using the scripts aggregate_metrics.py and extract_perf_timings.py. A detailed data dictionary documents all files, variables, and value definitions. Table 1 summarises the aggregated files, including their row counts and key attributes.

**Table 1:** Aggregated CSV files in the dataset deposit.

File	Rows	Key columns
all_rounds_flowertrained_mhealth.csv	220	session_id, variant, round, accuracy, loss, f1_macro, f1_weighted, precision_macro, recall_macro, specificity_macro, auc_macro, f1_class_0…11, round_wall_time_s, blockchain_blocks, model_payload_bytes, ipfs_model_cid, ipfs_local_cid, ipfs_vote_cid, ipfs_metrics_cid
all_rounds_flowertrained_ucihar.csv	220	session_id, variant, round, accuracy, loss, f1_macro, f1_weighted, precision_macro, recall_macro, specificity_macro, auc_macro, f1_class_0…5, round_wall_time_s, blockchain_blocks, model_payload_bytes, ipfs_model_cid, ipfs_local_cid, ipfs_vote_cid, ipfs_metrics_cid
all_devices_flowertrained_mhealth.csv	2000	session_id, variant, round, client_id, train_loss, num_examples, training_time_s, active_classes, cpu_percent, ram_used_mb, cpu_temp_c
all_devices_flowertrained_ucihar.csv	2000	session_id, variant, round, client_id, train_loss, num_examples, training_time_s, active_classes, cpu_percent, ram_used_mb, cpu_temp_c
all_client_evals_flowertrained_mhealth.csv	2000	session_id, variant, round, client_id, eval_loss, eval_acc, eval_f1, eval_auc, num_examples, eval_time_seconds, cpu_percent, ram_used_mb, cpu_temp_c
all_client_evals_flowertrained_ucihar.csv	2000	session_id, variant, round, client_id, eval_loss, eval_acc, eval_f1, eval_auc, num_examples, eval_time_seconds, cpu_percent, ram_used_mb, cpu_temp_c
perf_timings_flowertrained_mhealth.csv	2560	session_id, variant, round, event_type, block_type, duration_ms, extra_key, extra_value
perf_timings_flowertrained_ucihar.csv	2560	session_id, variant, round, event_type, block_type, duration_ms, extra_key, extra_value
session_summary_flowertrained_mhealth.csv	20	session_id, variant, dataset, num_rounds, final_accuracy, final_f1_macro, final_auc_macro, final_loss, peak_accuracy, mean_accuracy, peak_f1_macro, mean_round_wall_time_s, total_wall_time_s, num_samples, model_payload_bytes
session_summary_flowertrained_ucihar.csv	20	session_id, variant, dataset, num_rounds, final_accuracy, final_f1_macro, final_auc_macro, final_loss, peak_accuracy, mean_accuracy, peak_f1_macro, mean_round_wall_time_s, total_wall_time_s, num_samples, model_payload_bytes
tx_pin_map_flowertrained_mhealth.csv	840	run, variant, pinata_account, fl_round, block_type, expected_pin, pin_status, tx_hash, basescan_url, ipfs_hash, pinata_account_actual, date_pinned
tx_pin_map_flowertrained_ucihar.csv	840	run, variant, pinata_account, fl_round, block_type, expected_pin, pin_status, tx_hash, basescan_url, ipfs_hash, pinata_account_actual, date_pinned

Each ZIP archive contains 20 session directories: 10 baseline and 10 optimised sessions per benchmark. *Each directory is* named according to the middleware variant and execution timestamp. Each session directory includes the experiment configuration, training and evaluation results, server logs, a performance log file, and eleven confusion-matrix images corresponding to rounds 0–10.

The results.json files are stored in newline-delimited JSON (NDJSON) format and contain three types of records. Global records provide round-level server metrics, including accuracy, F1-scores, AUC, precision, recall, specificity, per-class metrics, confusion matrices, round duration, blockchain statistics, model size, and IPFS content identifiers. Device training records capture per-client training information such as loss, sample count, training time, CPU utilisation, memory usage, and temperature. Client evaluation records store per-client evaluation metrics, including loss, accuracy, F1-score, AUC, evaluation time, and device telemetry. The ordering of activity classes used in per-class metrics and confusion matrices is defined by the superclass_names field and documented in DATA_DICTIONARY.md.

The experiment_config.json file contains session-level metadata, including the middleware variant, timestamp, federated learning hyperparameters, IPFS configuration, hardware specifications, and session start time. The perf_<session_id>.log files record the timestamped latency of each blockchain and storage operation. The fl_server.log file captures the server-side execution log, while each cm_round_N.png file visualises the test-set confusion matrix for round N. The smart contract source code (FLBL2.sol) and compiled ABI (FLBL2_abi.json) are also provided in the GitHub repository.

**Fig. 1:**
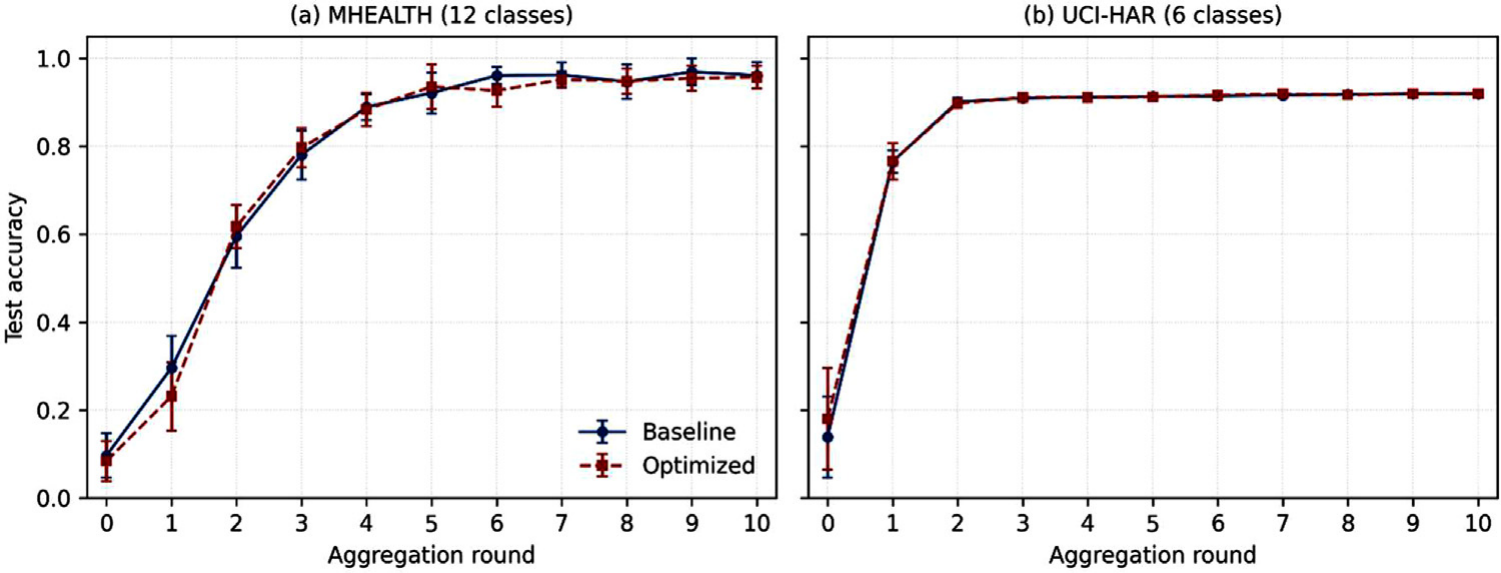
FL test-set accuracy over the ten aggregation rounds for the baseline and optimised variants: (a) MHEALTH; (b) UCI-HAR. Points are the mean across the 10 sessions per variant; error bars represent ±1 standard deviation.

**Fig. 2:**
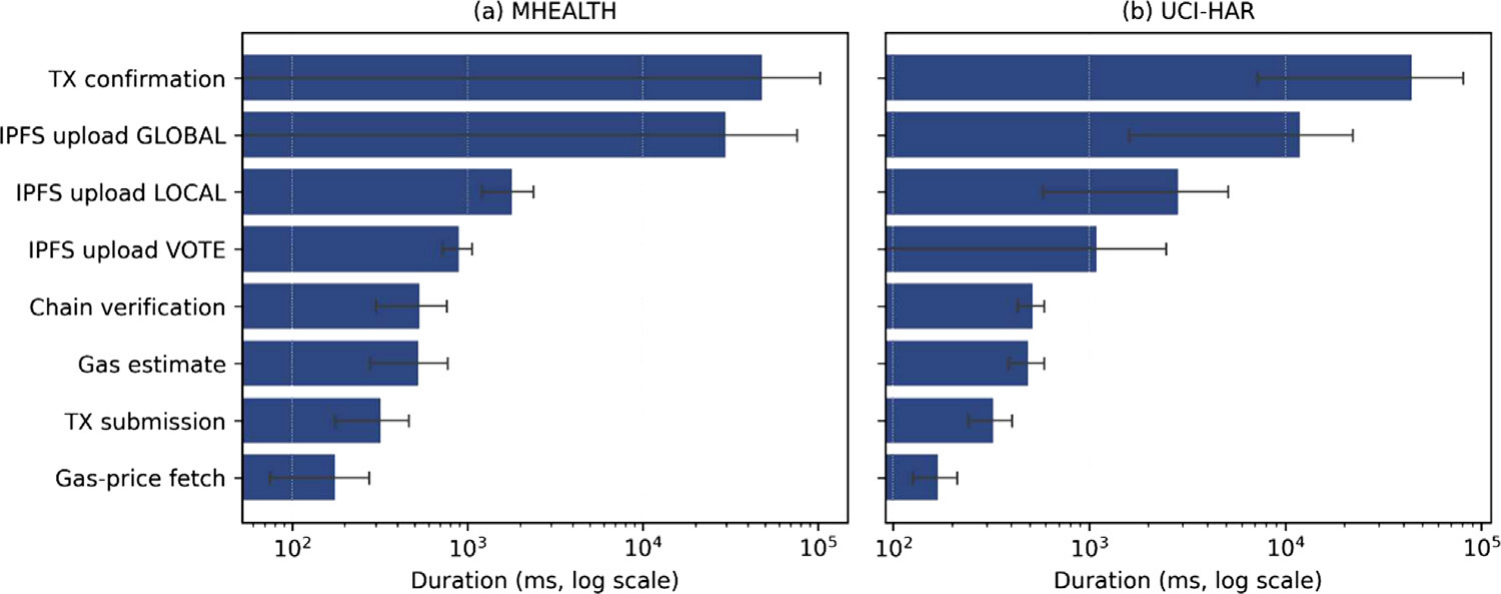
Mean latency (ms, log scale) of blockchain and IPFS operations for (a) MHEALTH and (b) UCI-HAR sessions. Error bars represent ±1 standard deviation; operation counts are reported in Table 2.

Table 2 summarises key statistics derived from the aggregated dataset, while Fig. 1 illustrates federated learning accuracy across aggregation rounds for both datasets and middleware variants. Fig. 2 presents the latency distributions of recorded blockchain and IPFS operations. The 243,917 ms stall noted in Table 2 occurred in round 6 of one optimised UCI-HAR session (optimized_20,260,626_190,710) and was caused by a delayed transaction confirmation on Base mainnet rather than an IPFS gateway timeout: the round's GLOBAL transaction was submitted normally but was confirmed only after 243,917 ms, landing roughly 150 blocks later than the round's other two transactions, while the round's IPFS uploads completed normally in the background. The event is recorded as event_type tx_confirm in perf_timings_flowertrained_ucihar.csv, so simulation studies can either retain it as a realistic tail-latency event of a public Layer-2 endpoint or exclude it as an outlier.

**Table 2:** Summary statistics computed from the aggregated CSV files (mean ± standard deviation). For UCI-HAR, round overhead is reported as the median because one optimised round recorded a 243,917 ms network stall; full per-event values are in perf_timings_flowertrained_ucihar.csv.

Metric	Scope	Baseline	Optimised
*MHEALTH (12 classes, 514 test windows, 23 channels at 50Hz)*			
Round-10 accuracy	10 sessions	0.962 ± 0.029	0.957 ± 0.026
Peak accuracy (any round)	10 sessions	0.986 ± 0.016	0.972 ± 0.021
Round-10 macro-F1	10 sessions	0.961 ± 0.030	0.956 ± 0.027
Round wall time (s)	100 rounds	96.0 ± 67.3	74.5 ± 28.5
Round overhead (ms)	110 rounds	1820 ± 740	1079 ± 510
IPFS upload GLOBAL (ms)	110 uploads	29,773 ± 46,166	—
IPFS upload LOCAL (ms)	100 uploads	1804 ± 580	—
IPFS upload VOTE (ms)	100 uploads	897 ± 174	—
Transaction confirmation (ms)	620 transactions	47,913 ± 55,248 (both variants)	
Gas estimate (ms)	310 / 30 records	529 ± 253	492 ± 193
Gas-price fetch (ms)	310 / 10 records	175 ± 102	171 ± 38
Device CPU utilisation (%)	2000 records	83.4 ± 0.4 (both variants)	
Device RAM use (MB)	2000 records	1047 ± 30 (both variants)	
Device CPU temperature ( °C)	2000 records	63.7 ± 3.1 (both variants)	
IPFS pin success	840 pins	97.1% (816/840)	
*UCI-HAR (6 classes, 2947 test windows, 9 channels at 50Hz)*			
Round-10 accuracy	10 sessions	0.920 ± 0.005	0.920 ± 0.005
Peak accuracy (any round)	10 sessions	0.922 ± 0.005	0.922 ± 0.005
Round-10 macro-F1	10 sessions	0.921 ± 0.005	0.920 ± 0.005
Round wall time (s)	100 rounds	80.0 ± 20.3	77.2 ± 34.3
Round overhead (ms)	110 rounds	1639 (median)	822 (median)
IPFS upload GLOBAL (ms)	110 uploads	11,819 ± 10,223	—
IPFS upload LOCAL (ms)	100 uploads	2845 ± 2265	—
IPFS upload VOTE (ms)	100 uploads	1094 ± 1376	—
Transaction confirmation (ms)	620 transactions	44,197 ± 36,922 (both variants)	
Gas estimate (ms)	310 / 30 records	493 ± 103	446 ± 97
Gas-price fetch (ms)	310 / 10 records	170 ± 43	154 ± 49
Device CPU utilisation (%)	2000 records	91.1 ± 1.2 (both variants)	
Device RAM use (MB)	2000 records	986 ± 50 (both variants)	
Device CPU temperature ( °C)	2000 records	69.4 ± 4.4 (both variants)	
IPFS pin success	840 pins	100.0% (840/840)	
*Combined totals*			
Total FL sessions		40	
Total FL aggregation rounds		400	
Total on-chain transactions		1240	
Total IPFS pins		1680

## 4 Experimental Design, Materials and Methods

### 4.1 Federated learning protocol

Training is performed using the Flower 1.29.0 [8] framework and the Federated Averaging (FedAvg) protocol [5]. Each session consists of ten aggregation rounds, beginning with an initial evaluation round (round 0) using the randomly initialised global model. In subsequent rounds, all ten clients participate, receiving the current global model, training locally on their private data (five local epochs for MHEALTH, three for UCI-HAR), and returning updated parameters and sample counts. The server then aggregates the client updates through equal-weight averaging, evaluates the resulting global model on a fixed test set, and records the round-level metrics and telemetry described in the dataset.

### 4.2 Model

The classifier is a one-dimensional Squeeze-and-Excitation residual network [9,10] with approximately 860,000 trainable parameters and a gzip-serialised size of 3439,144 bytes for MHEALTH (3420,320 bytes for UCI-HAR), which is bit-identical across rounds for a given session. The network applies four convolutional stages (channel widths 32, 64, 128, 256), each with batch normalisation, a rectified-linear activation, max-pooling, and squeeze-and-excitation residual blocks, followed by global average pooling, dropout (0.3), and a linear classification head. Local training minimises a focal loss (γ = 2) [11] to counter class imbalance, using the AdamW optimiser (*learning rate of 0.002 for MHEALTH and 0.001 for UCI-HAR, weight decay* 1 × 10⁻⁴) with cosine-annealing scheduling. Mixup (α = 0.3), additive Gaussian noise, and amplitude jitter are applied as augmentations during local training only.

### 4.3 Source datasets and client partitioning

Two public human activity recognition benchmarks [12,13] were used, although the raw datasets are not redistributed. MHEALTH contains data from 10 subjects performing 12 activities across 23 sensor channels sampled at 50 Hz. Signals were segmented into overlapping 256-sample windows, with subjects 1–8 assigned to training clients and subjects 9–10 reserved for testing. Local training used 5 epochs, a learning rate of 0.002, and a batch size of 64. UCI-HAR contains smartphone accelerometer and gyroscope data from 30 subjects performing 6 activities across 9 channels. Using the benchmark's standard train-test split, 21 subjects were distributed among training clients, and 9 subjects formed a fixed test set. Local training used 3 epochs, a learning rate of 0.001, and a batch size of 64.

### 4.4 Physical testbed

**Fig. 3:**
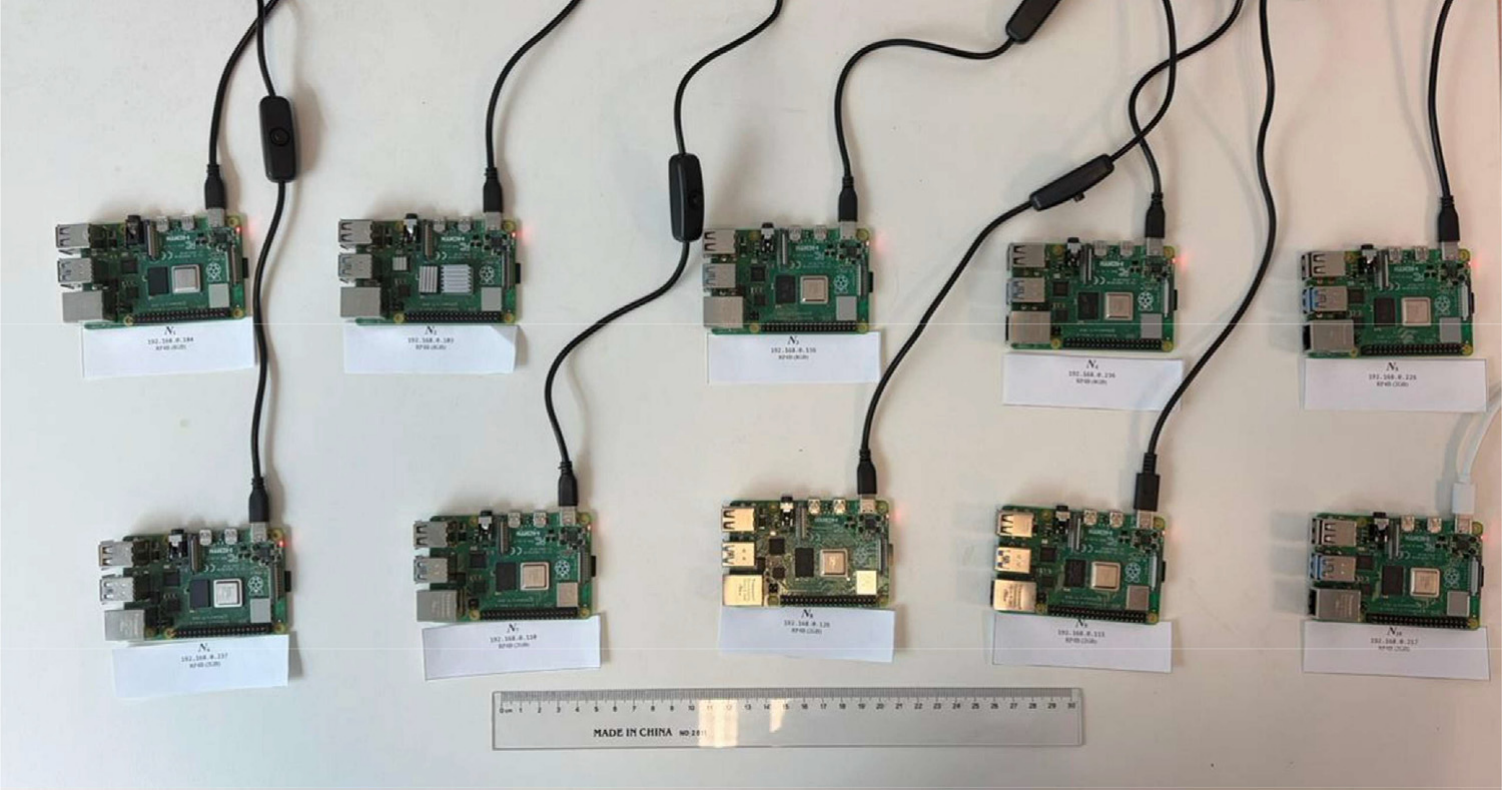
The physical federated-learning testbed: ten Raspberry Pi 4 Model B devices (nodes N₁ – N₁₀), each labelled with its local-network address and memory configuration (2 GB or 4 GB). A 30 cm ruler indicates scale.

**Fig. 4:**
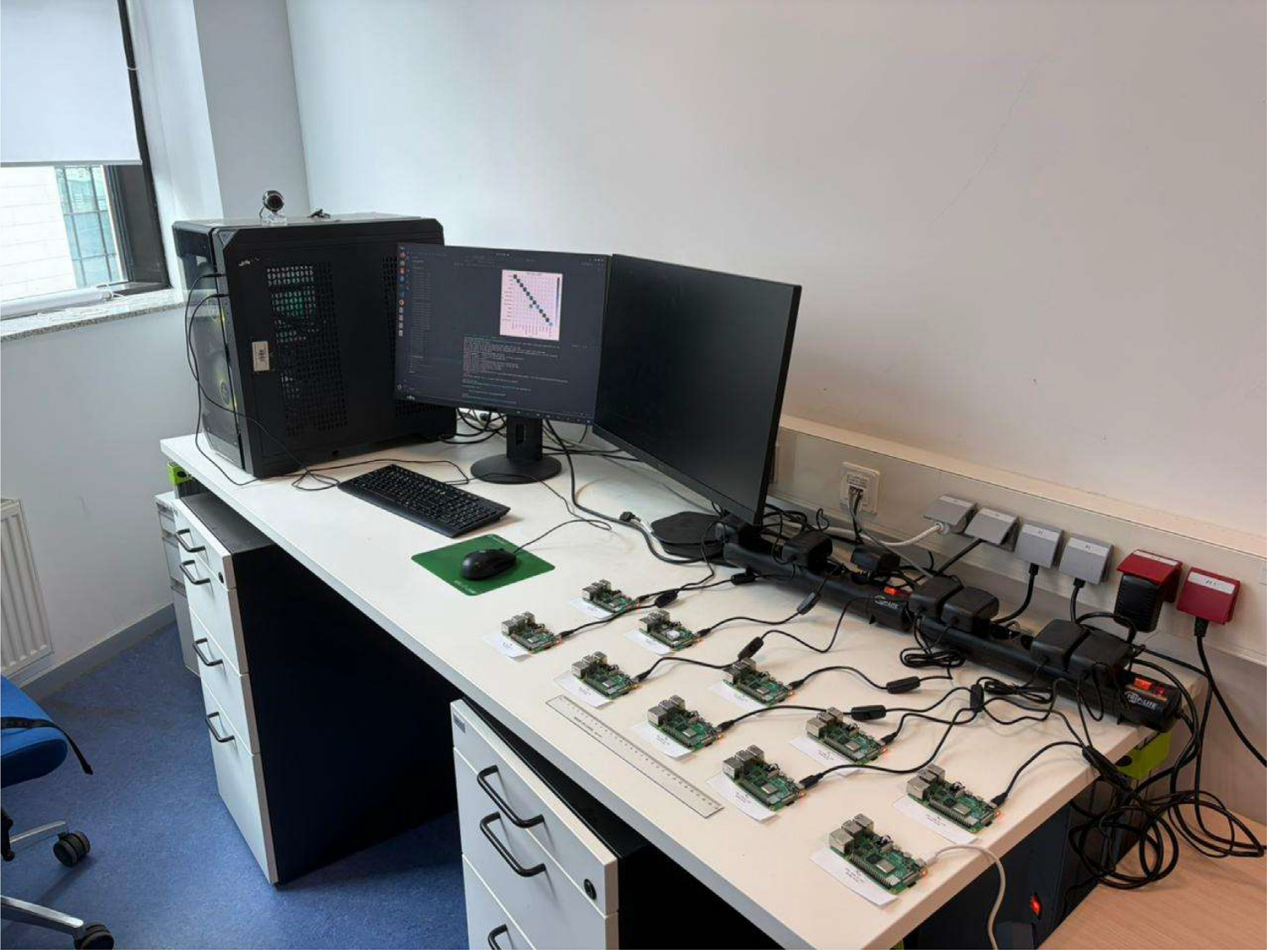
Wider view of the physical deployment: the ten Raspberry Pi 4 client nodes (N₁–N₁₀) together with the Ubuntu aggregation server that runs the Flower SuperLink, the web3.py blockchain middleware, and the Pinata pinning service. All devices share a single 2.4 GHz Wi-Fi network.

Ten Raspberry Pi 4 Model B single-board computers act as the federated clients (Fig. 3). Each runs Raspberry Pi OS 64-bit with Python 3.11 and PyTorch 2.7.1 on a quad-core ARM Cortex-A72 processor with 2 GB or 4 GB of LPDDR4 memory. The devices connect over a shared 2.4 GHz Wi-Fi access point on a private local network to the aggregation server, an Ubuntu workstation running the Flower SuperLink, the web3.py 7.x blockchain middleware, and the Pinata pinning API (Fig. 4). Sessions ran under sustained load for over nine hours without thermal throttling.

During both local training and evaluation, each client records device-level telemetry and reports it as part of the round data. CPU utilisation is calculated from process CPU time over the corresponding wall-clock interval and normalised by the number of processor cores. Memory usage is obtained from the system's virtual memory statistics, while device temperature is measured from the Raspberry Pi thermal sensor, with a fallback sensor library for other platforms. These measurements are stored in the cpu_percent, ram_used_mb, and cpu_temp_c fields of all records.

The ram_used_mb field records the absolute amount of system memory in use on the device, not a fraction of installed capacity. Because every client runs the same operating system image, software stack, and per-round training workload, this footprint is determined by the workload rather than by the installed RAM: per-client means span 979–1070 MB for MHEALTH and 911–1047 MB for UCI-HAR, with no systematic separation between the 2 GB and 4 GB devices. The footprint remains well below the 2 GB capacity of the smallest devices, so no client experienced memory pressure or swapping in either variant.

### 4.5 Blockchain infrastructure

The FLBL2 audit contract (Solidity 0.8.20, OpenZeppelin v5.0.2) is deployed on Base mainnet, an OP-Stack Ethereum Layer-2 (chain ID 8453), at 0xBE3FeAC76293711C5D32426860303AfE24cdf527, and is verified on Basescan; a public permissionless chain keeps every audit record independently verifiable by any party [14,15]. The contract initialises a genesis block at deployment and exposes addBlock(flRound, blockType, data), verifyChain(), and getBlockCount(). Each call to addBlock stores the round index, the block type, a keccak-256 content hash of the submitted payload, and a previous-block hash that links the new block to its predecessor, forming a hash chain whose integrity verifyChain() re-checks end to end [2,4]. Three block types are written: GLOBAL commits the identifiers of the aggregated model and round-metrics artifacts; LOCAL commits the identifier of the client-update summaries; and VOTE commits the identifier of the per-client loss votes used for informational anomaly annotation. Round 0 writes a single GLOBAL block; rounds 1–10 each write LOCAL, VOTE, and GLOBAL blocks, giving 31 on-chain records per session. Transaction submission and confirmation latencies, the landing block number, and per-round gas parameters were recorded for every transaction in all sessions. At the low Base Layer-2 gas prices observed during the experiment, with each transaction consuming a few hundred thousand gas units, the on-chain cost per round remained a small fraction of a US dollar [16].

Model and metric artifacts are stored off-chain using IPFS [17] and pinned through the Pinata service, while only their content identifiers (CIDs) are recorded on-chain, keeping blockchain storage requirements minimal regardless of artifact size. A SHA-256 hash is retained for each artifact to enable integrity verification after retrieval. In the baseline configuration, uploads are completed before transaction submission, whereas the optimised configuration overlaps IPFS uploads with local training using a background thread. Failed uploads are automatically retried, resulting in pinning success rates of 816/840 for MHEALTH and 840/840 for UCI-HAR.

### 4.6 Middleware variants and aggregation pipeline

Each benchmark was evaluated using two middleware configurations that produce identical model performance and on-chain records but differ in execution efficiency. The baseline variant performs all blockchain and storage operations synchronously and recomputes gas parameters every round. The optimised variant improves efficiency by caching gas-limit estimates and gas prices, overlapping IPFS uploads with local training through a background thread, and deferring blockchain verification until the end of the session. These optimisations substantially reduce blockchain-related overhead while preserving identical auditing and learning outcomes.

Each session writes a timestamped performance log that records the latency of blockchain and storage operations. Each entry includes the event time, operation type, duration, and optional metadata such as gas estimates, gas prices, block numbers, or verification results. Logged operations include IPFS uploads, gas estimation, gas-price retrieval, transaction submission, confirmation latency, blockchain verification, and total per-round overhead. In the optimised variant the IPFS upload runs in a background thread that overlaps local training, so its latency is hidden behind computation rather than logged as a separate synchronous upload event.

The deposited CSV tables are produced by two scripts that read the raw session directories without modifying any value. aggregate_metrics.py enumerates the session directories, parses each results.json line by line as newline-delimited JSON, and flattens the global, device-training, and client-evaluation records into the round, device, evaluation, and session-summary tables. Per-class arrays are expanded into indexed columns (f1_class_0…f1_class_N); the hyphenated raw key num-examples is normalised to num_examples, and the raw key training_time becomes training_time_s. Session summaries exclude round 0 from timing statistics, because round 0 stores the session-start timestamp in the round_wall_time_s field rather than a duration. extract_perf_timings.py parses the per-session performance logs into the timing table. The transaction–pin maps are unchanged from the per-run audit files. No value in any table is imputed or estimated.

## Limitations

The data come from a 10-node Raspberry Pi 4 testbed on one Wi-Fi network and one Base mainnet endpoint. Transaction-confirmation times depend on public-network conditions at the time of collection.

## Ethics Statement

The authors confirm compliance with the ethical requirements of *Data in Brief*. This work does not involve human participants, animal experiments, or social media data. The source datasets (MHEALTH and UCI-HAR) are publicly available and de-identified benchmarks; this repository contains only derived model outputs, aggregated performance metrics, device telemetry, and blockchain audit records, and does not redistribute any raw participant data.

## CRediT Author Statement

**Talgar Bayan:** Conceptualization, Methodology, Software, Investigation, Data curation, Visualization, Writing – original draft, Project administration. **Bibarys Mukhambetiyar:** Software, Validation, Data curation, Writing – review & editing. **Askar Boranbayev:** Validation, Resources. **Adnan Yazici:** Conceptualization, Supervision, Funding acquisition, Writing – review & editing.

## Data Availability

Mendeley DataNew Dataset of Performance Metrics, Device Telemetry, and Blockchain Audit Logs from a Federated Learning Testbed on Raspberry Pi Devices for HAR (Original data)
